# A SUMO-dependent pathway controls elongating RNA Polymerase II upon UV-induced damage

**DOI:** 10.1038/s41598-019-54027-y

**Published:** 2019-11-29

**Authors:** Irina Heckmann, Maximilian J. Kern, Boris Pfander, Stefan Jentsch

**Affiliations:** 1 Max Planck Institute of Biochemistry, Molecular Cell Biology, 82152 Martinsried, Germany; 2 Max Planck Institute of Biochemistry, DNA Replication and Genome Integrity, 82152 Martinsried, Germany

**Keywords:** Molecular biology, DNA damage and repair, Transcription

## Abstract

RNA polymerase II (RNAPII) is the workhorse of eukaryotic transcription and produces messenger RNAs and small nuclear RNAs. Stalling of RNAPII caused by transcription obstacles such as DNA damage threatens functional gene expression and is linked to transcription-coupled DNA repair. To restore transcription, persistently stalled RNAPII can be disassembled and removed from chromatin. This process involves several ubiquitin ligases that have been implicated in RNAPII ubiquitylation and proteasomal degradation. Transcription by RNAPII is heavily controlled by phosphorylation of the C-terminal domain of its largest subunit Rpb1. Here, we show that the elongating form of Rpb1, marked by S2 phosphorylation, is specifically controlled upon UV-induced DNA damage. Regulation of S2-phosphorylated Rpb1 is mediated by SUMOylation, the SUMO-targeted ubiquitin ligase Slx5-Slx8, the Cdc48 segregase as well as the proteasome. Our data suggest an RNAPII control pathway with striking parallels to known disassembly mechanisms acting on defective RNA polymerase III.

## Introduction

DNA is the macromolecule that harbors all information required for life. In eukaryotes three nuclear RNA polymerases (RNAPI–III) are necessary to read out this information. While RNAPI transcribes ribosomal RNA (rRNA)^1^, RNAPIII mediates transcription of rRNA, transfer RNAs (tRNA) and other small RNAs^2^. RNAPII is important for transcription of protein-coding genes, as well as for synthesis of non-coding RNAs^3–5^.

All three RNA polymerases are under control of several posttranslational modifications. However, specifically transcription by RNAPII is regulated by phosphorylation that chiefly targets Rpb1’s carboxyl-terminal domain (CTD) heptapeptide repeats (consensus sequence YSPTSPS)^6^, which serve as platform for factors involved in transcription, chromatin modification, mRNA splicing and export^7,8^. Phosphorylation of serine-5 (S5P) of the CTD repeats appears to be largely specific for Rpb1 molecules localized to promoters^9,10^. Serine-2 phosphorylation (S2P) in contrast is a hallmark for elongating polymerases and ChIP signals using S2P-specific antibodies steadily rise downstream of transcription start sites and culminate at the 3’-ends of genes^9,11–13^.

Highly problematic for all RNA Polymerases are bulky DNA lesions on the coding strand as they may behave as “roadblocks” leading to persistent stalling of the progressing protein complexes. A key mechanism to remove bulky DNA adducts and to avoid cell cycle arrest and cell death is nucleotide excision repair (NER)^14,15^. Yet to conduct DNA repair the machinery first needs to gain access to the lesion that is masked by a stalled polymerase and here, several possibilities exist. First, the RNA polymerase can simply be released from the DNA to allow access. This has been shown to occur for RNAPI and RNAPII, but the underlying mechanism is poorly understood^16–18^. Second, RNAPII backtracking from the lesion can facilitate repair without dissociation. This is made possible by the proofreading activity of RNAPII, which allows the complex to backtrack during transcription^19,20^. After RNAPII backtracking and successful DNA repair, reactivation of transcription requires RNA transcript cleavage for repositioning of the 3′-end with the active center of RNAPII^21,22^. Finally, active removal or even degradation of the stalled polymerase may serve as a temporary relief and perhaps facilitate DNA repair^23–25^.

Previous work has demonstrated that in cells exposed to UV light, which leads to pyrimidine dimers and 6-4 photoproducts in DNA, Rpb1, the largest subunit of RNAPII, becomes short-lived through degradation by the ubiquitin-proteasome system (UPS)^26^. By monitoring the entire population of Rpb1 using Rpb1-specific antibodies, it was initially shown that Rpb1 is ubiquitylated by the ubiquitin ligase Rsp5, which binds to the CTD of Rpb1^27–31^. Other studies found that Rpb1 ubiquitylation is mediated by an Elongin-Cul3-dependent ubiquitin ligase complex (Cul3-Roc1-Elc1-Ela1)^32,33^. Later, it was suggested that the two distinct ubiquitin ligases act within a sequential pathway^23,34^. In the current model, Rpb1 is first mono-ubiquitylated or modified by a lysine-63 (K63)-linked poly-ubiquitin chain. The de-ubiquitylation enzyme Ubp2 removes Rsp5-dependent K63-linked poly-ubiquitin chains to mono-ubiquitylation, allowing the Elongin-Cul3 ligase to add K48-linked poly-ubiquitin chains that induce Rpb1 degradation^34^. Another de-ubiquitylation enzyme, Ubp3, was suggested to reverse both Rpb1 poly- and mono-ubiquitylation^35^. Removal of K48-linked poly-ubiquitylated Rpb1 from damaged DNA is furthermore dependent on the ATPase Cdc48^36^. To this end, Cdc48 cooperates with the adaptor proteins Ubx4/Ubx5 to mediate extraction of Rpb1 from the chromatin-bound RNAPII complex and subsequent delivery to the proteasome for degradation^36,37^.

Apart from the above-mentioned negative regulation by ubiquitin, the catalytic core domain of Rpb1 is also modified with SUMO, which requires the SUMO ligase Siz1 together with the SUMO-conjugating enzyme Ubc9^38,39^. SUMOylation of Rpb1 is induced upon DNA damage or transcriptional impairment, but has not been linked to Rpb1 ubiquitylation or proteasomal degradation. Instead, this modification is thought to restrain DNA damage signaling induced by a stalled RNAPII complex^39^.

In this study, we revealed a further layer of RNAPII regulation upon DNA damage in the budding yeast *Saccharomyces cerevisiae*. Focussing on the transcription-engaged pool of RNAPII we discovered a SUMO-dependent mechanism, which specifically controls the serine-2 phosphorylated (S2P) form of RNAPII. Upon UV treatment, we observed rapid SUMOylation of Rpb1-S2P and subsequent decrease of both S2P- and SUMO-modified Rpb1. Disappaerance of S2P-modified Rpb1 is dependent on the SUMO-targeted ubiquitin ligase Slx5-Slx8, the ubiquitin/SUMO- dependent segregase Cdc48 and the proteasome.

## Results and Discussion

### S2-phosphorylated Rpb1 declines upon DNA damage induction via a mechanism involving the proteasome and Cdc48 segregase

**Figure 1:**
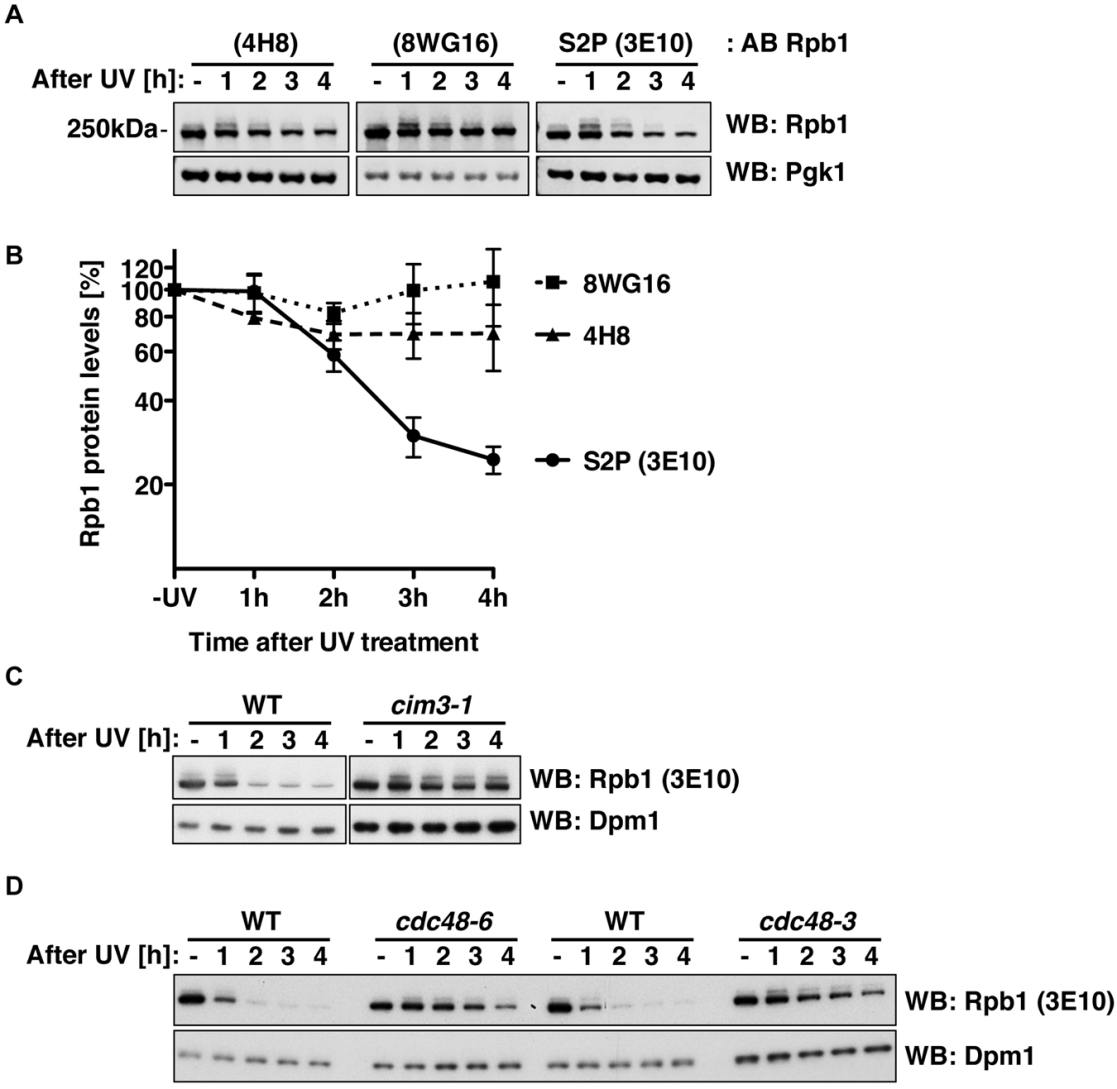
S2-phosphorylated Rpb1 disappears upon DNA damage induction via a mechanism involving the proteasome and the segregase Cdc48. (**A**) Levels of Rpb1 and modified forms in WT cells treated with UV irradiation (400 J/m^2^) followed by a recovery time course in YPD media in the dark, which blocks repair by photolyase. For the western blots (WB) antibodies against the C-terminal domain (CTD) of Rpb1 were used. 3E10 recognizes phosphorylated serine-2 in the CTD^9,13,41^, 4H8 and 8WG16 recognize the CTD independently of any modification^82,83^. Pgk1 served as loading control. (**B**) Quantification of Rpb1 signals as in (**A**). Quantification was performed on LI-COR Odyssey with normalization to Pgk1. Data represent mean ± standard deviation calculated from four biological replicates, presented as relative amount to the untreated (-UV) sample. (C + D) Rpb1-S2P levels in WT, proteasome mutant *cim3-1* (**C**) and *cdc48-6* or *cdc48-3* (**D**) cells after UV irradiation (400 J/m^2^) followed by a recovery time course in YPD media. Cells were shifted to 37 °C for 1 h before irradiation and for the recovery phase. For the western blots (WB) anti-Rpb1 (3E10) antibody was used. Dpm1 served as loading control.

The fate of transcriptionally stalled RNAPII upon DNA damage has been investigated previously^26,28–30,34^. Degradation of the RNAPII largest subunit Rpb1 has been shown using antibodies recognizing the entire Rpb1 pool (such as monoclonal antibodies 4H8 and 8WG16)^26,34,40^. As the fate of elongating RNAPII, which would collide with DNA lesions is particularly relevant, we aimed to monitor the S2-phosphorylated form as a proxy for elongating RNAPII (monoclonal antibody 3E10^9,40–42^). To assay RNAPII levels, we challenged budding yeast cells with an acute dose of UV light (400 J/m^2^) and probed samples at different time points after irradiation by western blotting using the different antibodies. Using antibodies against the entire pool of Rpb1 (4H8 and 8WG16), we observed a moderate loss of Rpb1 signals over 4 hours indicating degradation^26,27,34,43^ (Fig. 1A, left and central panel and Fig. 1B). When we used the S2P-specific antibody (3E10) to probe these samples, we observed that the Rpb1 S2P signal started to decrease 2 h after UV treatment and dropped almost by fivefold within 4 h after UV treatment (Fig. 1A, right panel and Fig. 1B). In contrast, UV treatment had no discernable effect on the Rpb1-S5P signal (Fig. S1A, right panel). Although we followed the same Western Blot densiometry protocol in all experiments, it needs to be noted that effects observed with different antibodies might be difficult to compare in quantitative manner. Nonetheless, we found striking that specifically the elongating, S2-phosphorylated form of RNAPII underwent strong changes after UV treatment.

To further analyze whether the drop in Rpb1-S2P levels was due to protein turnover, we treated cells with the protein synthesis inhibitor cycloheximide with or without additional induction of UV damage. While in cycloheximide-treated cells the Rpb1-S2P turnover was moderate (Fig. S1B, left panel), the Rpb1-S2P signal dropped much faster, when a combination of CHX and UV treatment was used (Fig. S1B, right panel). A UV dose of 400 J/m^2^ is lethal for the majority of cells, but UV-induced reduction in the Rpb1-S2P signal occurred in a dose-dependent manner over a wide range of UV doses (50 J/m^2^ – 400 J/m^2^, Fig. S1C–E).

We also used the UV-mimetic compound 4-nitroquinoline 1-oxide (4NQO)^44^, which induces DNA damage through reactive oxygen species that is also repaired by nucleotide excision repair. Also treatment of yeast cells with 20 µg/ml 4NQO caused a decrease in the Rpb1-S2P signal (Fig. S1F).

Previous work has demonstrated that exposure to UV or 4NQO leads to degradation of Rpb1 by the ubiquitin-proteasome system (UPS)^26,27,34^. Therefore we wanted to clarify, whether the observed loss of the Rpb1-S2P signal was also dependent on the proteasome. Indeed, the Rpb1-S2P signal remained stable when proteasome mutant *cim3-1* cells were treated with UV light (Fig. 1C). Moreover, when we tested for involvement of the ubiquitin/SUMO-dependent segregase Cdc48^36,45,46^, we found that the Rpb1-S2P signal was stabilized in UV-challenged *cdc48-6* and *cdc48-3* mutant cells (Fig. 1D). Overall, these data suggest that the elongating, S2-phosphorylated pool of RNAPII is diminished after DNA damage in a manner that depends on the proteasome and Cdc48.

### UV damage triggers Ubc9-, Siz1- and Siz2-dependent SUMOylation of Rpb1

**Figure 2:**
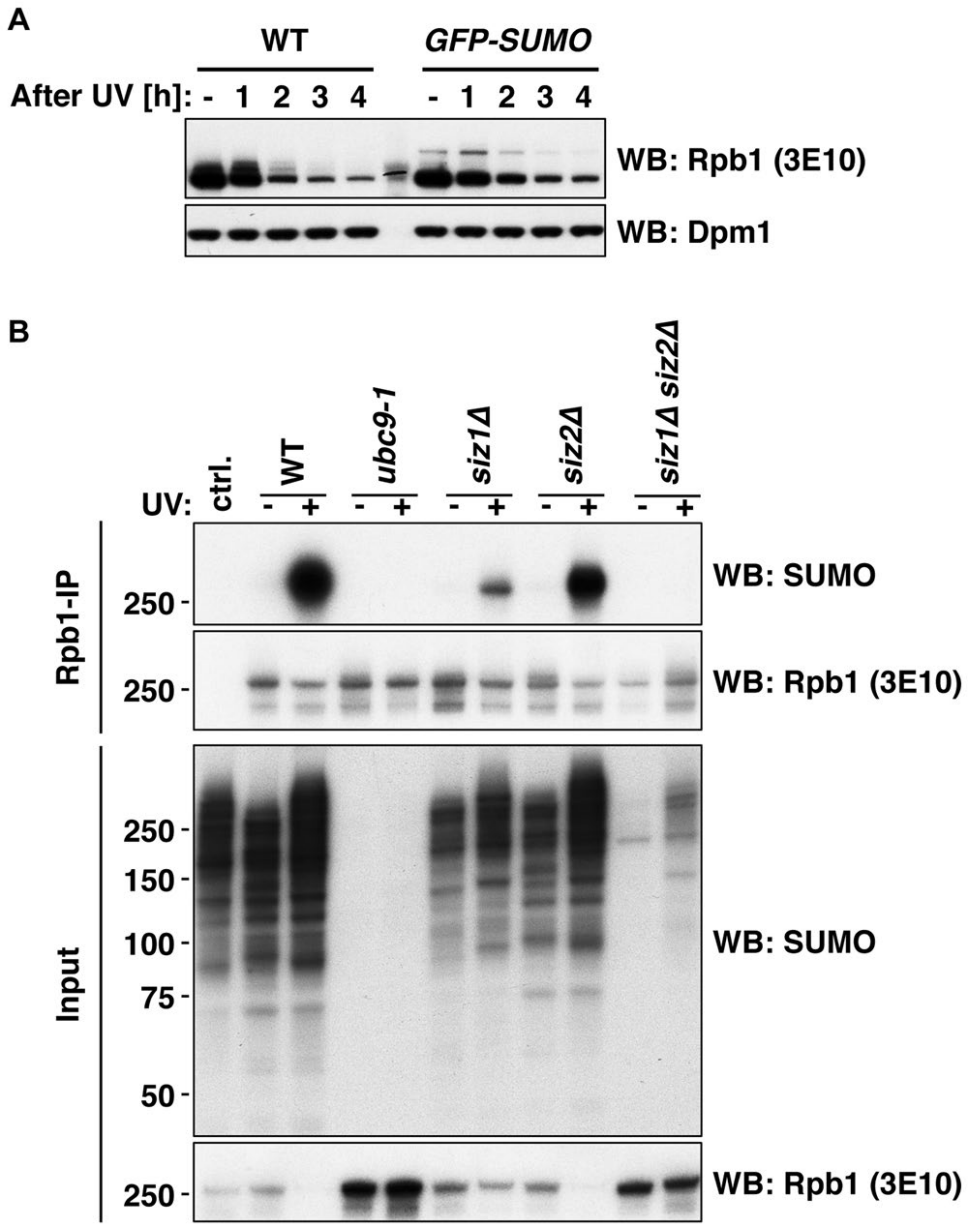
Rpb1 of elongating RNAPII is SUMOylated upon UV treatment. (**A**) Rpb1-S2P and the corresponding SUMO-modified form in WT cells and cells expressing GFP-tagged SUMO after UV irradiation (400 J/m^2^) followed by a recovery time course in YPD medium. SUMOylation is indicated by mobility shift of Rpb1-S2P with the tagged SUMO protein. The 3E10 antibody was used to detect Rpb1. Dpm1 served as loading control. (**B**) Immunoprecipitation of Rpb1 with Rpb1-S2P-specific antibody (3E10) from UV-treated and untreated WT, *ubc9-1*, *siz1Δ*, *siz2Δ* and *siz1Δ siz2Δ* double mutant cells. SUMOylated species of Rpb1 were detected by western blotting (WB) using SUMO-specific antibody.

After UV irradiation, we consistently observed a slower migrating Rpb1 species that reacted with all Rpb1 antibodies used, suggesting that this was a post-translationally modified version of Rpb1. Levels of this modified Rpb1 species were especially pronounced at early time points after UV exposure (Fig. 1A). Several RNAPII subunits were previously reported to be SUMO substrates^47,48^ and Rpb1 specifically becomes SUMOylated^38,39,48,49^. We therefore tested whether the observed slower migrating species is a SUMOylated form of Rpb1. We expressed a variant of SUMO (Smt3 in yeast) fused with an N-terminal GFP-tag (*GFP-SUMO*). Expression of GFP-SUMO caused an upshift of the slower migrating species of Rpb1 (Fig. 2A), suggesting it was indeed SUMO-modified Rpb1. To confirm this finding, we also probed immunoprecipitated Rpb1 from unchallenged and UV-treated cells by western blotting with a SUMO-specific antibody and found that the upshifted Rpb1 species after UV damage contained SUMO (Fig. 2B, WT + UV).

Next, we introduced mutations into the SUMO pathway. Using mutants of the SUMO-conjugating enzyme Ubc9 and the SUMOligases (Siz1 and Siz2), we corroborated previous findings^39,48^ and found that Rpb1 SUMOylation is mediated by Ubc9, Siz1, and to a lesser extent by Siz2 (Fig. 2B, right panel). However, we still observed Rpb1 SUMOylation when we mutated a proposed target lysine residue (K1487)^39^, as well as several other potential target sites^31^ to non-SUMOylatable arginine residues (Fig. S2). These data are consistent with the idea that SUMO may target multiple lysine residues of Rpb1, as has been seen for other SUMO substrates^47,49^.

### Serine-2 phosphorylated Rpb1 is regulated by a SUMO-dependent pathway

**Figure 3:**
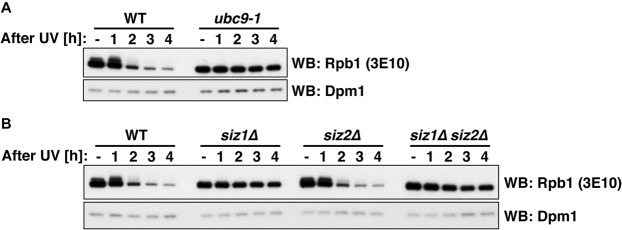
The SUMO conjugating system is required for UV-induced loss of S2-phosphorylated Rpb1. (A + B) Levels of Rpb1-S2P in WT and *ubc9-1* cells (**A**) and *siz1Δ*, *siz2Δ* and *siz1Δ siz2Δ* double mutant cells (**B**) after UV irradiation (400 J/m^2^). In (**A**) cells were shifted to 37 °C for 1 h before irradiation followed by a recovery in YPD media at 37 °C. The 3E10 antibody was used to detect Rpb1 by western blotting (WB). Dpm1 served as loading control.

To investigate whether UV-induced Rpb1 SUMOylation and the decline of the Rpb1-S2P signal are related, we investigated UV-induced loss of S2-phosphorylated Rpb1 in SUMO pathway mutant cells. Strikingly, the Rpb1-S2P signal was stable in mutants defective in Ubc9 or Siz1 even after UV irradiation (Fig. 3A,B**)**. In contrast, *SIZ2-*deficient cells showed loss of the Rpb1-S2P signal, similar to WT cells (Fig. 3B**)**. The same set of enzymes, which are responsible for UV-induced Rpb1 SUMOylation are therefore required for UV-induced disappearance of S2-phosphorylated Rpb1. Overall, these data are consistent with several models: (i) the SUMO-dependent mechanism acts on regulators of elongating RNAPII like the kinases relevant for S2 phosphorylation. (ii) UV-induced SUMOylation of Rpb1 triggers removal of stalled RNAPII from chromatin or renders it otherwise resistant to S2 phosphorylation. (iii) The SUMO-dependent mechanism leads to degradation of Rpb1 from stalled RNAPII complexes.

To address the first model, we tested whether the kinases that mediate CTD S2 phosphorylation of Rpb1^13,50,51^ are degraded after UV damage. We used antibodies against Ctk1^52^ or Bur1^53^ and followed respective protein levels after UV treatment (Fig. S3A,B). Interestingly, however, we found that endogenous protein levels of Ctk1 and Bur1 did not change significantly after UV treatment, while Rpb1-S2P levels dropped (Fig. S3A,B). Therefore, the S2-phosphorylating kinases are apparently not regulated by UV, at least not on protein level. Yet, these experiments do not exclude that SUMO and ubiquitin regulate the S2-phosphorylated pool of Rpb1 by other means.

### SUMO-targeted ubiquitin ligases (STUbLs) control RNAPII after UV damage

**Figure 4:**
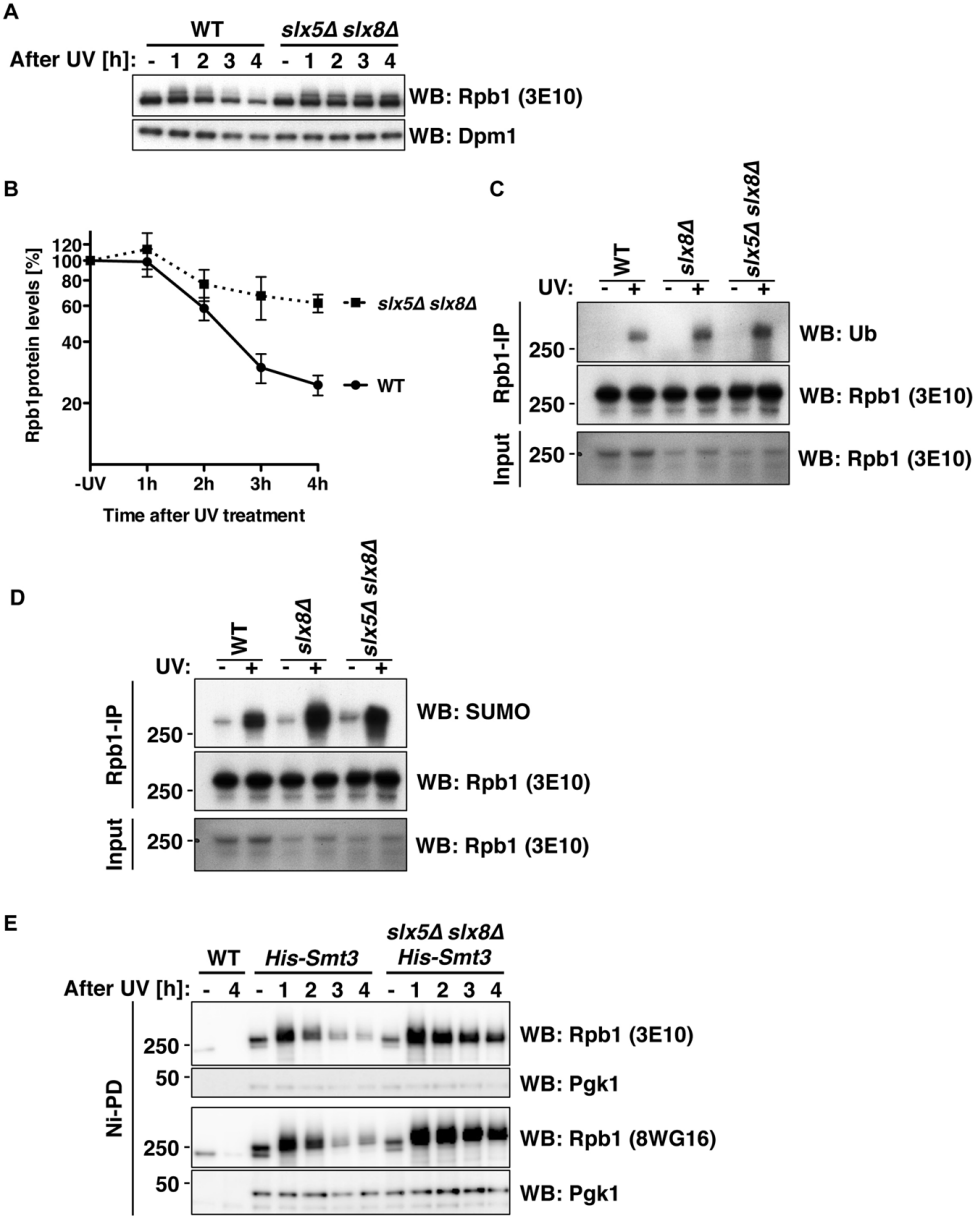
The STUbL Slx5-Slx8 regulates the SUMOylated pool of Rpb1. (**A**) Levels of Rpb1-S2P in WT and *slx5Δ slx8Δ* cells after UV irradiation (400 J/m^2^). The 3E10 antibody was used to detect Rpb1 by western blotting (WB). Dpm1 served as loading control. (**B**) Quantification of Rpb1-S2P levels in WT and STUbL-deficient *slx5Δ slx8Δ* cells as described in (**A**). Quantification was performed on a LI-COR Odyssey system with normalization to Pgk1. Shown are mean ± standard deviation calculated from four biological replicates, presented as relative amount compared to the untreated (-UV) sample. (C + D) Immunoprecipitation of Rpb1 with Rpb1-S2P-specific antibodies (3E10) from UV-treated and untreated WT, *slx8Δ* and *slx5Δ slx8Δ* cells. Ubiquitylated (**C**) and SUMOylated (**D**) species of Rpb1 were detected by western blotting (WB) using ubiquitin- and SUMO-specific antibodies succesively on the same blot. Therfore, control blots for Rpb1 are the same for (C) and (D). (**E**) Denaturing Ni-NTA pulldowns (Ni-PD) were performed to isolate His-SUMO-conjugates from UV-treated WT and *slx5Δ slx8Δ* cells following recovery in YPD. SUMOylated species of Rpb1 were detected by western blotting (WB) using 3E10 (Ser2P-CTD) or 8WG16 (CTD) antibodies. SUMOylated Pgk1 served as pulldown control.

Similar to our findings, also RNA polymerase III was recently found to be a SUMOylation target and RNAPIII SUMOylation was found to be induced when RNAPIII transcription was defective^54,55^. Interestingly, SUMO modification led to RNAPIII ubiquitylation by the heterodimeric SUMO-targeted ubiquitin ligase (STUbL) Slx5-Slx8^55^. This ubiquitin-modification was found to induce Cdc48-dependent extraction of RNAPIII from chromatin and downregulation of RNAPIII transcription, most likely by proteasomal degradation^55,56^. Given the similarities to UV-induced regulation of RNAPII-S2P, we hypothesized that also RNAPII might be regulated by Slx5-Slx8. Indeed, we found that cells deficient in Slx5-Slx8 did not show a reduction in the Rpb1-S2P signal after UV-induced DNA damage, but rather the Rpb1-S2P signal remained stable (Fig. 4A,B).

We therefore asked whether Rpb1 is a ubiquitylation substrate of Slx5-Slx8 and probed Rpb1-S2P immunoprecipitated from unchallenged and UV-treated cells with ubiquitin-specific antibodies. UV-induced ubiquitylation of Rpb1-S2P was readily detectable (Fig. 4C**)**. Notably, however, this ubiquitin modification was apparently unrelated to Slx5-Slx8 function, as we still observed an Rpb1-S2P ubiquitin signal in cells lacking *SLX8* or both *SLX5* and *SLX8* (Fig. 4C). In contrast, similar to published data^28,32,33^, we found Rpb1 ubiquitylation to depend on the ubiquitin ligases Rsp5 and Elongin-Cul3 (Fig. S4A,B). Specifically, we probed for ubiquitylation of immunoprecipitated Rpb1-S2P using ubiquitin-specific antibodies after UV treatment and found it to be defective in a temperature-sensitive mutant of *RSP5* (*rsp5-1*) (Fig. S4A**)** and in cells lacking subunits of the Elongin-Cul3 ligase (*elc1Δ*, *ela1Δ* and *cul3Δ*) (Fig. S4B). Moreover, the Rsp5-dependent ubiquitylation signal does also not rely on the SUMO-conjugating enzyme Ubc9 or the SUMO ligases Siz1 and Siz2 (Fig. S4C,D). Overall, we thereby confirmed previous reports, showing that Rpb1 is ubiquitylated involving both Rsp5 and the Elongin-Cul3 ligase complex^27,32,33^, but this ubiquitylation signal is apparently unrelated to the SUMO-STUbL pathway. We therefore conceded the assay was unable to visualize STUbL-dependent ubiquitylation of Rpb1, suggesting that either (i) STUbL-dependent ubiquitylation of Rpb1 is too short-lived to be detected in our IP-based assay, (ii) that STUbLs target RNAPII on subunits other than Rpb1, similar to RNAPIII, where the primary ubiquitylation target of Slx5-Slx8 is Rpc160^55^ or (iii) that STUbLs do not target RNAPII at all, but rather a protein involved in RNAPII phosphorylation or transcription elongation in general.

In either of the first two scenarios, SUMO-modified Rpb1-S2P could be seen as an intermediate on the way to ubiquitylation and this intermediate would be expected to accumulate in the absence of STUbLs. Consistent with this idea, we detected an accumulation of the SUMOylated form of Rpb1-S2P when we treated cells lacking *SLX8* or both *SLX5* and *SLX8* with UV (Fig. 4D). To further support these data, we enriched SUMOylated proteins using denaturing Ni-NTA pulldown of His-SUMO conjugates. SUMOylated proteins from WT and STUbL-deficient cells before UV treatment and at different time points thereafter were enriched and probed with the Rpb1 S2P-specific antibody (3E10) or the whole pool antibody (8WG16) (Fig. 4E). In line with our previous findings, SUMOylated species of Rpb1 peaked one hour after UV treatment and dropped gradually thereafter. In *SLX5-SLX8*-deficient cells, however, loss of SUMOylated Rpb1 species was minor and delayed, independent of the antibody used for Rpb1 detection (Fig. 4E). Overall, we therefore conclude that Rpb1-SUMOylation and loss of the Rpb1-S2P signal are connected, either because (i) Rpb1-SUMO and Slx5-Slx8 mediate ubiquitylation and chromatin extraction of Rpb1 or (ii) Rpb1-SUMO and Slx5-Slx8 regulate another factor, which critically controls the phosphorylation of RNAPII or perhaps transcription elongation itself.

Our data therefore reveal an unexpected function of SUMO and the STUbL Slx5-Slx8 in the regulation of RNAPII after UV-induced DNA damage. Our genetic and biochemical data point to several possible molecular mechanisms that could be responsible for loss of Rpb1-S2P.

First, Rpb1-S2P loss might be caused by STUbL-dependent Rpb1 ubiquitylation and degradation. This model would be entirely consistent with the involvement of the proteasome and the STUbL-dependent stabilization of SUMOylated Rpb1. However, when testing with IPs we did not observe evidence of STUbL-dependent Rpb1-S2P ubiquitylation. Rpb1 might therefore not be a direct target for Slx5-Slx8-dependent ubiquitylation. Alternatively, the modification might be very transient, as it is often observed for proteolysis-inducing ubiquitylation marks^55,57,58^. Notably, we could confirm Rsp5- and Elongin-Cul3 complex-dependent ubiquitylation on Rpb1^28,32,33^. One possible scenario is therefore that Rpb1 is targeted by different kinds of ubiquitin modifications. In this scenario the Rsp5/Elongin-Cul3-dependent ubiquitylation would not lead to degradation of the S2-phosphorylated polymerase and is therefore detectable in IP experiments while the Slx5-Slx8-dependent ubiquitylation would lead to degradation of the S2-phosphorylated polymerase and is therefore very transient. Overall, the relation between Rsp5/Elongin-Cul3 and STUbL-dependent mechanisms needs further clarification.

Second, Rpb1-S2P loss could come from turnover of the entire RNAPII complex, not just the Rpb1 subunit. SUMOylated Rpb1 might serve as a recruiting factor for the STUbL Slx5-Slx8, but then target RNAPII subunits other than Rpb1 would become ubiquitylated, potentially explaining why no STUbL-dependent ubiquitylation was detected for Rpb1. STUbL-dependent ubiquitylation of proteins that are in complex with the SUMOylated STUbL-recruitment factor have been suggested in other cases and importantly for RNAPIII^55,59,60^ giving precedence for this model. In contrast, previous work has shown that specifically Rpb1, but not other subunits become destabilized upon UV treatment^61^.

Third, UV-induced regulation of the Rpb1 kinases/phosphatases could lead to Rpb1-S2P loss. The major Rpb1-CTD S2 kinases are Ctk1 and Bur1, which belong to the cyclin-dependent kinase (CDK) family^6,13,62^. Besides RNAPII, they target also other factors to regulate several aspects of transcription^63–65^ and translation^52^. Particularly, histone ubiquitylation and SUMOylation were shown to prevent Ctk1 recruitment to chromatin and subsequent Rpb1-S2 phosphorylation^66,67^. Histone SUMOylation could therefore be involved in SUMO-dependent downregulation of Rpb1-S2 after UV, but current data do not explain the involvement of Slx5-Slx8. Other possible ways how Ctk1 and Bur1 could be regulated after UV could involve the CDK-activating kinase Cak1^68,69^ or the Fcp1 phosphatase, which dephosphorylates the CTD at serine 2^50^.

Fourth, Rpb1-S2P loss might simply reflect removal of RNAPII from chromatin by the Cdc48 segregase and regulation in a non-degradative fashion. However, this hypothesis in itself does not explain the involvement of the proteasome mediating decrease of the Rpb1-S2P signal. An involvement of the proteasome would therefore have to be indirect. A speculative scenario could be, that in proteasome mutants, extraction of RNAPII is linked to Hpr1, a member of the THO/TREX complex involved in mRNA export. Hpr1 is known to be regulated by the proteasome^70,71^. Degradation of Hpr1 after mRNA export is crucial to release chromatin-bound RNAPII^72^. Hpr1 was also shown to be SUMOylated, but SUMOylation does not appear to affect its stability, but rather interactions with mRNPs^73^.

Overall, although the precise mechanism by which STUbLs and the SUMO pathway regulate RNAPII after UV-induced DNA damage will need to be further clarified by future research, we now add another layer of regulation of RNAPII upon transcriptional stalling. Our data suggest that RNAPII is controlled by the sequential action of the small ubiquitin-like modifier (SUMO) and ubiquitin. Such a sequential mechanism allows the opportunity to differentially recruit proteins containing ubiquitin-and/or SUMO-interacting motifs. For instance, Cdc48 together with its co-factor Ufd1  were shown to target both ubiquitylated and SUMOylated proteins and, strikingly, also STUbL substrates^46,60,74^. SUMOylation of stalled RNAPII might therefore be a first line of defense and help in recruiting repair factors. Prolonged stalling may require additional means and involve Slx5-Slx8 and Cdc48, perhaps to segregate RNAPII from chromatin similar to its role in the release of chromatin-associated proteins or complexes^55,60,75,76^.

## Materials and Methods

### Yeast strains

Yeast strains used in this study are listed in Table S1.

### Yeast techniques and cloning

Yeast strains used in this study were constructed using standard techniques^77,78^. All strains are isogenic to DF5 (except for FW1808, which is derived from FY56^79^) and have the genotypes as described in Table S1. Yeast His-tagged SUMO was expressed from the integrative vector YIplac211 under the control of the *ADH1* promoter. Rpb1 point mutant variants were constructed using standard mutagenesis PCR techniques and expressed from the centromeric YCplac111 under the endogenous *RPB1* promoter.

### UV-C treatment of yeast cells

Yeast cells were grown to an OD_600_ of 0.5 at 30 °C in YPD media, after which they were optionally incubated for 1 h at 37 °C to induce temperature-sensitive phenotypes. Otherwise, cells were grown to an OD_600_ of 1 at 30 °C. About 50 OD_600_ of cells were harvested and washed once with PBS. Cells (50 OD_600_) were pelleted, washed once with PBS and irradiated in 140 mm culture dishes (BS04-irradiation chamber, Dr. Gröbel UV-Elektronik GmbH) in 40 ml PBS buffer. The cell suspension was pelleted in a dark falcon tube (5 min, 500 *g*) and resuspended in 30 ml YPD. To follow UV-recovery, cells were incubated for 4 h at 30 °C or 37 °C while shaking. For protein analysis, samples of 0.8 OD_600_ cells were harvested every hour, pelleted, frozen in liquid nitrogen and stored at −80 °C. For cell survival analysis, cells were plated on YPD plates after 4 hours of recovery and cell colonies were counted after 2 days.

### Protein analysis

Total cellular protein extracts were prepared by TCA precipitation^77^, separated using NuPAGE Bis-Tris 4–12% gradient gels (Life Technologies) and analyzed by standard western blotting techniques.

### Immunoprecipitation of Rpb1 and Ni-NTA pulldown

For Rpb1-immunoprecipitation, about 50 OD_600_ cells were resuspended in lysis buffer (50 mM HEPES pH 7.5, 1 mM EDTA, 1% Triton X-100, 0.1% Na-deoxycholate, 10 mM NEM, and complete protease inhibitors). Cell lysis was performed with a beadshaker (MM301, Retsch) using silica beads. Afterwards, cell lysates were sonicated (water bath sonification: Bioruptor UCD-200, Diagenode) and centrifuged (21,000 *g*, 10 min). The extract was incubated with Rpb1 antibodies (3E10 or 8WG16) for 1.5 h. Protein A Sepharose (GE healthcare) was added for 30 min. Incubation steps were performed on a rotation wheel at 4 °C. The beads were pelleted, washed with lysis buffer (3-times) and boiled in Laemmli buffer.

Denaturing Ni-NTA pulldowns were described previously^80^. About 50 OD_600_ of cells were harvested, frozen in liquid nitrogen and stored at −80 °C. The pellets were resuspended in cold lysis buffer (1.85 M NaOH, 7.5% β‐mercaptoethanol) to a final volume of 6 ml and incubated for 15 min on ice. An equal volume of 55% trichloroacetic acid (TCA) was added, followed by incubation for 15 min on ice. Proteins were pelleted (3.500 *g*, 5 min, 4 °C), washed twice with cold water and resuspended in 12 ml of buffer A and 0.05% Tween-20 (buffer A: 6 M guanidinium chloride, 100 mM NaH_2_PO_4_, 10 mM Tris; pH 8.0). For resolubilzation, the suspensions were transferred into centrifuge tubes and incubated (1 h, 180 rpm) while shaking. The clear supernatant was collected (20 min, 4 °C, 23,000 *g*), imidazole (final concentration 20 mM) and 200 *µ*l of Ni-NTA agarose slurry (Qiagen) were added to the tubes. The suspension was incubated overnight (4 °C). The agarose slurry was washed with 50 ml of buffer A plus 0.05% Tween 20, and 50 ml of buffer C plus 0.05% Tween 20 (buffer C: 8 M urea, 100 mM NaH_2_PO_4_, 10 mM Tris/HCl; pH 6.3). For protein elution, 1 ml of elution buffer (buffer C, 0.05% Tween-20, 250 mM imidazole) was added to the Ni‐NTA column. The eluted proteins were precipitated with 1 ml of 55% TCA, incubated for 10 min and centrifuged (20,000 *g*, 30 min). The pellet was boiled in 30 µl HU-Buffer for 10 min at 65 °C.

### Antibodies

Rpb1 antibodies 3E8 (Millipore), 3E10 (Millipore), 8WG16 (Abcam) and 4H8 (Abcam) were used. Anti-SUMO (Smt3)^81^ and Bur1^53^ antibodies were described before. Anti-ubiquitin antibody P4D1 (Santa Cruz Biotechnology) and anti-Ctk1 (Abcam) were used. Anti-Dpm1 and anti-Pgk1 antibodies were purchased from Life Technologies.

### Reproducibility

Rpb1 stability experiments with a western blot read-out in Fig. 1A were performed in four or more biological replicates. Quantification results in Fig. 1B were calculated from four biological replicates. Rpb1 stability experiments in Fig. 1C were performed in three biological replicates. In Fig. 1D, Rpb1 stability experiments in *cdc48-6* cells were performed three times, in *cdc48-3* cells once. Rpb1 stability experiments in Fig. 2A were performed twice. In Fig. 2B, Rpb1 SUMOylation in *ubc9-1* cells was tested in two biological replicates, in *siz1Δ* cells in four biological replicates, in *siz2Δ* cells in two biological replicates and in *siz1Δ siz2Δ* cells in three biological replicates. Rpb1 stability experiments of Fig. 3A,B were performed in two and three biological replicates, respectively. Rpb1 stability experiments in Fig. 4A were performed in four or more biological replicates. Quantification results in Fig. 4B were calculated from four biological replicates. Experiments to detect Rpb1 ubiquitylation or SUMOylation in Fig. 4C,D were performed in three biological replicates. Denaturing Ni-PDs in Fig. 4E were performed in three biological replicates.

## Supplementary Information

Supplementary information
