# Supplementary material for: A SUMO-dependent pathway controls elongating RNA Polymerase II upon UV-induced damage
Source: Sci Rep. 2019 Nov 29;9:17914. doi: 10.1038/s41598-019-54027-y (PMC6884465; doi:10.1038/s41598-019-54027-y)
Supplement: Supplementary file 1 — Supplementary information [file 41598_2019_54027_MOESM1_ESM.pdf]

A SUMO-dependent pathway controls elongating RNA Polymerase II upon UV-induced damage

Irina Heckmann[1], Maximilian J. Kern[1], Boris Pfander[2*] and Stefan Jentsch[1#]

Affiliations:

[1] Max Planck Institute of Biochemistry, Molecular Cell Biology, 82152 Martinsried, Germany.

[2] Max Planck Institute of Biochemistry, DNA Replication and Genome Integrity, 82152 Martinsried, Germany.

[#] Deceased during the course of this study

* Corresponding author:

e-mail: bpfander@biochem.mpg.de

Supplementary Information

Supplementary Material
Supplementary Figure Legends
Supplementary References
Supplementary Figures S1-S4

Supplementary Material

Table S1 Yeast strains used in this study.

Strain	Genotype	Reference
DF5	*his3Δ200 leu2-3,112 lys2-801 trp1-1 ura3-52*	[1]
YIH516	*elc1::hphNT1*	This study
MJK110	*ela1::hphNT1*	This study
MJK108	*cul3::hphNT1*	This study
FW1808	*his4-912 ΔR5, lys2-128Δ, ura3-52, rsp5-1*	[2]
Y0554	*cim3-1, ura3-52, leu2Δ1*	[3]
Y0649	*cdc48-6*	[4]
Y3784	*cdc48-3*	This study
MJK369	*pADH-GFP-Smt3::natNT2*	This study
Y0174	*ubc9Δ::TRP1 leu2::ubc9Pro-Ser::LEU2*	[5]
Y1558	*siz1::HIS3MX6*	This study
Y3619	*siz2::HIS3MX4*	This study
Y3061	*siz1::HIS3MX6 siz2::HIS3MX4*	This study
YIH443	*slx5::HIS3MX6 slx8::hphNT1*	This study
MJK116	*rpb1::hphNT1 YCplac111-rpb1::LEU*	This study
MJK117	*rpb1::hphNT1 YCplac111-rpb1K330R::LEU*	This study
MJK151	*rpb1::hphNT1 YCplac111-rpb1K217R::LEU*	This study
MJK155	*rpb1::hphNT1YCplac111rpb1K1487R::LEU*	This study
YIH607	*rpb1::hphNT1 YCplac111-rpb1K695R::LEU*	This study
YIH590	*YIplac211-pADH-His-Smt3-tADH::URA3*	This study
YIH599	*slx5::natNT2 slx8::hphNT1 YIplac211-pADH-Smt3-tADH::URA3*	This study

Reproducibility

Rpb1 stability experiments with a western blot read-out in Fig S1A, Fig S1B and Fig S1C were performed in tree biological replicates. Quantification results in Fig S1D were calculated from three biological replicates. Survival rate in Fig S1E was calculated from two biological replicates and three technical replicates each. Rpb1 stability experiment in Fig S1F was performed once. Experiments to detect Rpb1 ubiquitylation in Fig S2 were performed in two biological replicates. Rpb1 and Ctk1 or Bur1 stability experiments in Fig S3A and Fig S3B were performed in three biological replicates. Experiments to detect Rpb1 ubiquitylation in Fig S4 were performed in three biological replicates.

Supplementary References

1. Ulrich, H. D. & Jentsch, S. Two RING finger proteins mediate cooperation between ubiquitin-conjugating enzymes in DNA repair. *EMBO J.* **19,** 3388–3397 (2000).
2. Wang, G., Yang, J. & Huibregtse, J. M. Functional domains of the Rsp5 ubiquitin-protein ligase. *Mol. Cell. Biol.* **19,** 342–352 (1999).
3. Piwko, W. & Jentsch, S. Proteasome-mediated protein processing by bidirectional degradation initiated from an internal site. *Nat Struct Mol Biol* **13,** 691–697 (2006).
4. Bergink, S. *et al.* Role of Cdc48/p97 as a SUMO-targeted segregase curbing Rad51-Rad52 interaction. *Nat. Cell Biol.* **15,** 526–532 (2013).
5. Seufert, W., Futcher, B. & Jentsch, S. Role of a ubiquitin-conjugating enzyme in degradation of S- and M-phase cyclins. *Nature* **373,** 78–81 (1995).
6. Coordes, B. *et al.* Ctk1 function is necessary for full translation initiation activity in Saccharomyces cerevisiae. *Eukaryotic Cell* **14,** 86–95 (2015).
7. Clausing, E. *et al.* The transcription elongation factor Bur1-Bur2 interacts with replication protein A and maintains genome stability during replication stress. *J. Biol. Chem.* **285,** 41665–41674 (2010).

Supplementary Figure Legends

Fig S1 Elongating RNAPII is downregulated in a UV dose-dependent manner and upon 4NQO treatment

(A) Levels of Rpb1-S2P and -S5P in WT cells after UV irradiation (400 J/m^2). For the western blots (WB), antibodies against the C-terminal domain (CTD) of Rpb1 were used. 3E10 recognizes phosphorylated serine-2 in the CTD. 3E8 recognizes phosphorylated serine-5 in the CTD. Dpm1 served as loading control.

(B) Levels of Rpb1-S2P in untreated or UV-treated (400 J/m^2) WT cells followed by a recovery phase in YPD supplemented with cycloheximide (CHX) (100 μg/ml). For the WB, anti-Rpb1 (3E10) antibody was used. Dpm1 served as loading control.

(C) Levels of Rpb1-S2P in WT cells after different doses of UV irradiation (50-400 J/m^2) followed by a recovery time course in YPD media supplemented with cycloheximide (CHX) (100 μg/ml). For the WB, anti-Rpb1 (3E10) antibody was used. Pgk1 served as loading control.

(D) Quantification of Rpb1-S2P levels from experiment performed in (C). Quantification was performed on a LI-COR Odyssey system with normalization to Pgk1. Data represent mean ± standard deviation calculated from three biological replicates, presented as relative amount to untreated (-UV) sample.

(E) Survival rate of WT cells after different doses of UV irradiation (50-400 J/m^2) followed by a recovery time course of 4 hours in YPD medium as measured by colony forming units. Data are mean ± standard deviation calculated from two biological replicates (three technical replicates each), presented as relative amount compared to untreated (0 J/m^2) cells.

(F) Levels of Rpb1-S2P in untreated (0 μg/ml) or 4-NQO (20 μg/ml) treated cells. For the WB anti-Rpb1 (3E10) antibody was used. Dpm1 served as loading control.

Fig S2 SUMOylation of Rpb1 is not restricted to previously identified lysine residues

Immunoprecipitation of Rpb1 with Rpb1-S2P-specific antibody (3E10) from UV treated and untreated WT, *rpb1K217R*, *rpb1K1487R*, *rpb1K330R* and *rpb1K695R* cells. SUMOylated species of Rpb1 were detected by western blotting (WB) using SUMO-specific antibody.

Fig S3 Major Rpb1 S2 kinases are not down regulated upon UV treatment

(A+B) WT cells after UV irradiation (400 J/m^2). The 3E10 antibody was used to detect Rpb1. Endogenous levels of Ctk1 (A) and Bur1 (B) were detected with an anti-Ctk1[6] and anti-Bur1[7] antibody. Dpm1 levels served as loading control.

Fig S4 Rsp5 and Elongin-Cul3 ubiquitin ligases are critical for Rpb1 ubiquitylation

(A+B) Immunoprecipitation of Rpb1 with Rpb1-S2P-specific antibody (3E10) from UV-irradiated (+) and untreated (-) WT*, rsp5-1* (A), and *elc1Δ*, *ela1Δ*, *cul3Δ* (B) cells. Ubiquitylated species of Rpb1 were detected using the P4D1 ubiquitin-specific antibody. Sepharose beads without addition of the first antibody served as background-binding control (ctrl.).

(C+D) Immunoprecipitation of Rpb1 with Rpb1-S2P-specific antibody (3E10) from UV-irradiated (+) and untreated (-) WT*, ubc9* (A), and *siz1Δ siz2Δ* (B) cells. Ubiquitylated and SUMOylated species of Rpb1 were detected using the P4D1 ubiquitin-specific antibody and the SUMO-specific antibody, respectively. Sepharose beads without addition of the first antibody served as background-binding control (ctrl.)

Figure S1

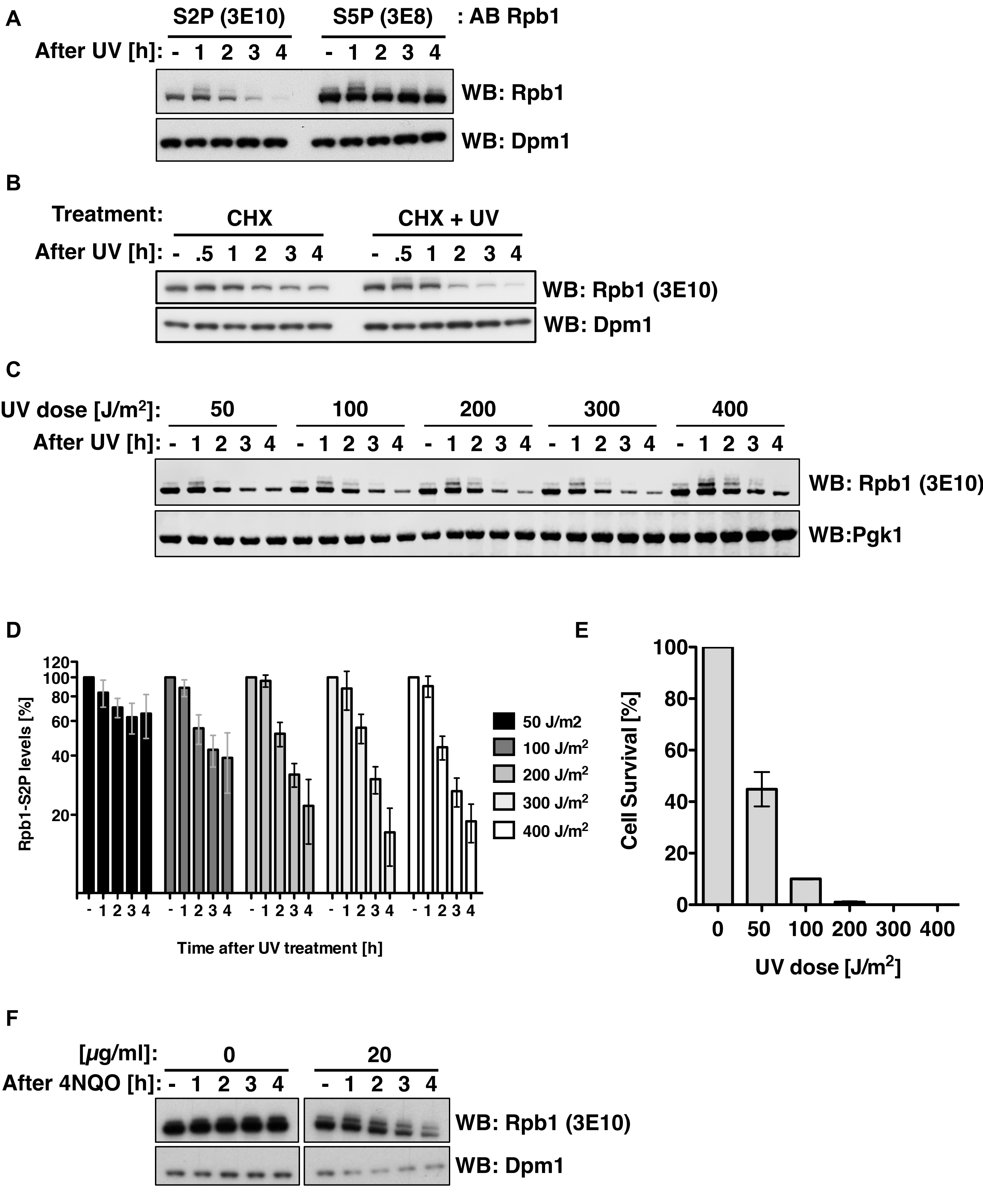

A
S2P (3E10)
S5P (3E8)
: AB Rpb1
After UV [h]: - 1 2 3 4 - 1 2 3 4
WB: Rpb1
WB: Dpm1
B
Treatment:
CHX
CHX + UV
After UV [h]: - .5 1 2 3 4 - .5 1 2 3 4
WB: Rpb1 (3E10)
WB: Dpm1
C
UV dose [J/m2]: 50 100 200 300 400
After UV [h]: - 1 2 3 4 - 1 2 3 4 - 1 2 3 4 - 1 2 3 4 - 1 2 3 4
WB: Rpb1 (3E10)
WB:Pgk1
D
Rpb1-S2P levels [%]
120
100
80
60
40
20
- 1 2 3 4
Time after UV treatment [h]
50 J/m2
100 J/m2
200 J/m2
300 J/m2
400 J/m2
E
Cell Survival [%]
100
80
60
40
20
0
0 50 100 200 300 400
UV dose [J/m2]
F
[μg/ml]: 0 20
After 4NQO [h]: - 1 2 3 4 - 1 2 3 4
WB: Rpb1 (3E10)
WB: Dpm1

Figure S2

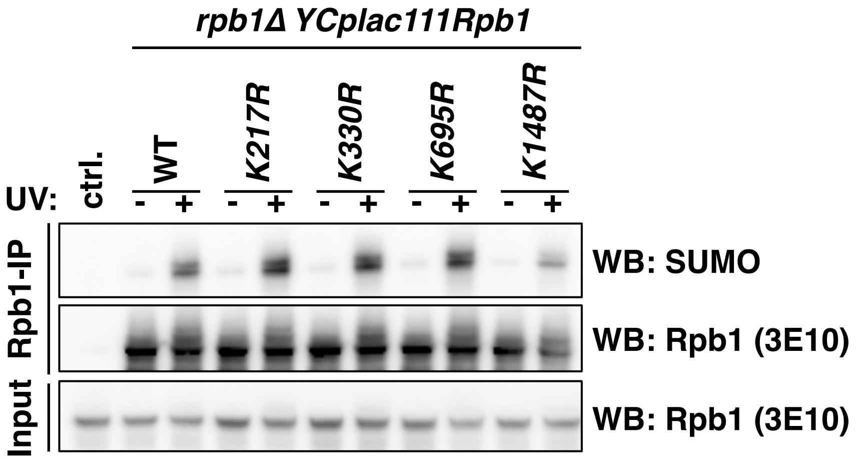
rpb1Δ YCplac111Rpb1
ctrl.
WT
K217R
K330R
K695R
K1487R
UV:
- + - + - + - + - +
Rpb1-IP
Input
WB: SUMO
WB: Rpb1 (3E10)
WB: Rpb1 (3E10)

Figure S3

A

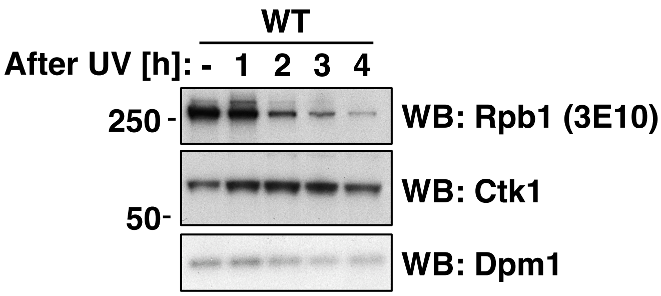
WT
After UV [h]: - 1 2 3 4
250
WB: Rpb1 (3E10)
WB: Ctk1
50
WB: Dpm1

B

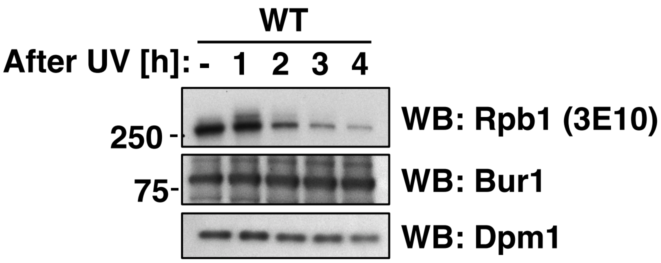
WT
After UV [h]: - 1 2 3 4
250
WB: Rpb1 (3E10)
75
WB: Bur1
WB: Dpm1

Figure S4

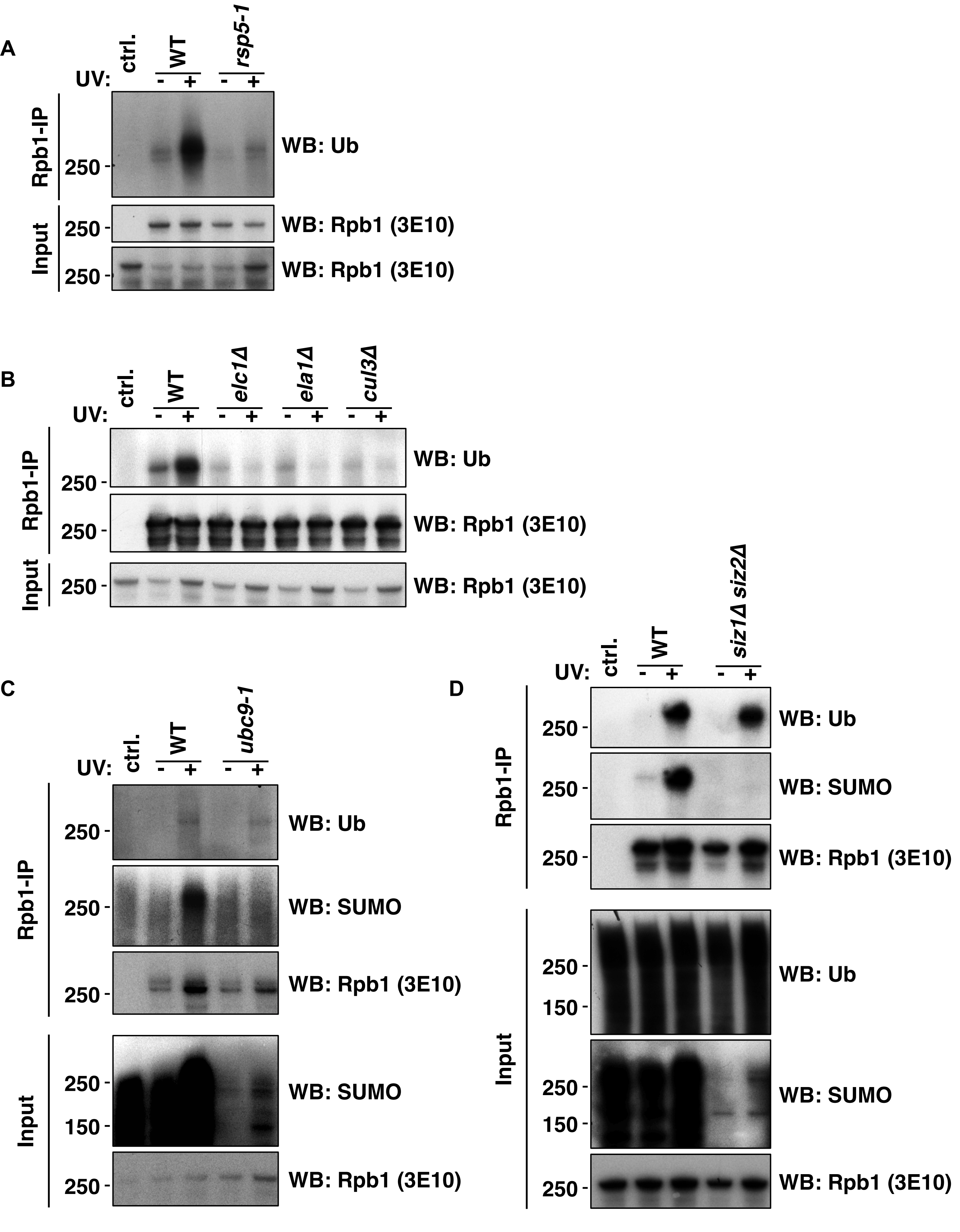
A
ctrl.
WT
rsp5-1
UV:
- + - +
Rpb1-IP
Input
250
WB: Ub
WB: Rpb1 (3E10)
WB: Rpb1 (3E10)
B
ctrl.
WT
elc1Δ
ela1Δ
cul3Δ
UV:
Rpb1-IP
Input
250
WB: Ub
WB: Rpb1 (3E10)
WB: Rpb1 (3E10)
C
ctrl.
WT
ubc9-1
UV:
Rpb1-IP
Input
250
150
WB: Ub
WB: SUMO
WB: Rpb1 (3E10)
WB: SUMO
WB: Rpb1 (3E10)
D
ctrl.
WT
siz1Δ siz2Δ
UV:
Rpb1-IP
Input
250
150
WB: Ub
WB: SUMO
WB: Rpb1 (3E10)
WB: Ub
WB: SUMO
WB: Rpb1 (3E10)
